# Supplementary material for: Singular sublimation of ice and snow crystals
Source: Nat Commun. 2018 Oct 10;9:4191. doi: 10.1038/s41467-018-06689-x (PMC6180084; doi:10.1038/s41467-018-06689-x)
Supplement: Supplementary file 1 — Supplementary Information [file 41467_2018_6689_MOESM1_ESM.pdf]

Supplementary Informations for "Singular sublimation of ice and snow crystals"

Etienne Jambon-Puillet, Noushine Shahidzadeh, Daniel Bonn
Institute of Physics, Van der Waals-Zeeman Institute, University of Amsterdam, Science Park 904, 1098 XH Amsterdam, the Netherlands

SUPPLEMENTARY NOTE 1: SHORT TIME TIP SMOOTHING MODEL

We model the tip of our drops as half of a two sheet hyperboloid of revolution whose equation is given in cylindrical coordinates by $z^2/a^2 - r^2/b^2 = 1$. The initial cone of semi angle α is drawn by the asymptotes of the projected hyperbola such that $b/a = \tan\alpha$ (see Supplementary Figure 1A) and the sharpness of the tip is given by its curvature $\kappa = a/b^2$ (the two principal curvatures are equal at the tip $\kappa_1 = \kappa_2 = \kappa$ [1]). The rest of the drop sits below the tip and the full vapor field $\rho(\mathbf{r})$ is a combination of the hyperboloidal tip field and the drop field. Since the gradient of the field is very strong close to the tip and decays very fast, we use a boundary layer approach similar to the one developped for Taylor cones [2] to compute the field (Supplementary Figure 1B).

In the boundary layer, Laplace's equation (1) is conveniently solved around the hyperboloidal tip with prolate spheroidal coordinates (ξ, η, ϕ)[3] since surfaces of constant ξ are hyperboloids. The general solution is $\rho = C_1 + C_2 \ln\left[(1+\xi)/(1-\xi)\right]$ with C_1 and C_2 integration constants [1, 2, 4]. The first boundary condition is $\rho(\xi_s) = \rho_{sat}$ where ξ_s represents the hyperboloid surface. The remaining constant is equal to the value of the field on the horizontal plane were the asymptotes meet ($\xi = 0$) that we consider as the edge of the boundary layer (Supplementary Figure 1B). The field around the hyperboloid is finally given by

$$\rho = \rho_{\xi=0} + (\rho_{sat} - \rho_{\xi=0}) \frac{\ln\left(\frac{1+\xi}{1-\xi}\right)}{\ln\left(\frac{1+\xi_s}{1-\xi_s}\right)}$$

and the flux at the tip surface by

$$j_n = \frac{D}{\rho_{ice}} \left.\frac{\partial \rho}{\partial n}\right|_{\xi_s} = \frac{D}{\rho_{ice} c} \sqrt{\frac{1-\xi^2}{\eta^2 - \xi^2}} \left.\frac{\partial \rho}{\partial \xi}\right|_{\xi_s} = -\frac{2D}{\rho_{ice} \ln\left(\frac{1+\xi_s}{1-\xi_s}\right)} \frac{\rho_{sat} - \rho_{\xi=0}}{c\sqrt{(\eta^2 - \xi_s^2)(1-\xi_s^2)}} \tag{1}$$

with n denoting the normal direction, $\frac{\partial}{\partial n} = \mathbf{n} \cdot \nabla$ the normal derivative and $c = \sqrt{a^2 + b^2}$ the linear eccentricity of the projected hyperbola. To close the problem we need to match this solution with the outer field to know $\rho_{\xi=0}$.

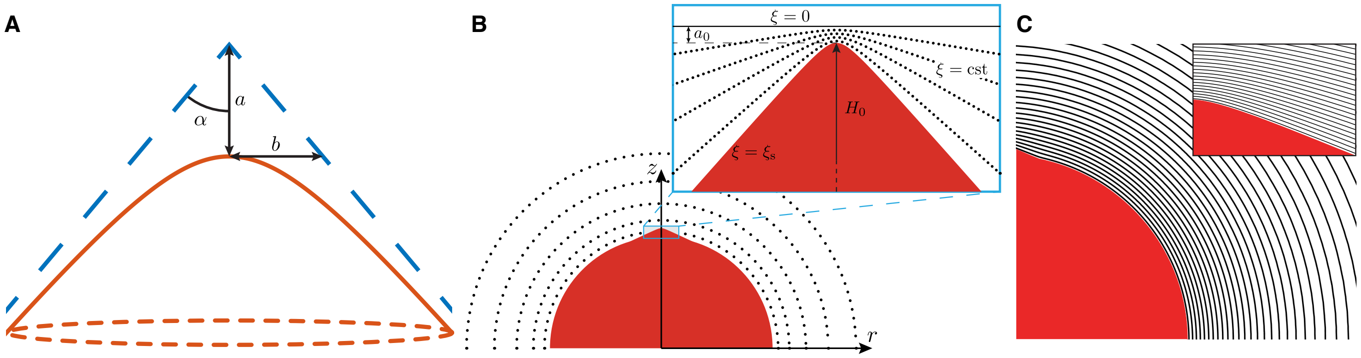

Supplementary Figure 1. **Hyperboloidal tip smoothing model. A** Schematic of the tip of the hyperboloid with its conical asymptotes. The cone semi-angle α and the parameters defining the hyperboloid a and b are drawn ($\tan\alpha = b/a$). **B** Schematic of the model: an hyperboloidal tip sits on a hemispherical drop. Far from the tip, the outer field is given by the hemisphere and must match the inner tip field at the edge of the boundary layer. The tip corresponds to the surface $\xi = \xi_s$, the dotted lines are equipotentials ($\xi = $ cst) and the solid line is the equipotential at the edge of the boundary layer: the horizontal plane $\xi = 0$ corresponding $z = H_0 + a_0$. **C** Full field for this geometry solved numerically. The lines represent equipotentials.

Matching with an hemisphere: For simplicity we first assume that the drop below the tip is a hemisphere. The outer field is thus $\rho - \rho_\infty = (\rho_{\rm sat} - \rho_\infty)R/\sqrt{r^2+z^2}$ and neglecting its curvature we have on the $\xi = 0$ or $z = H_0 + a_0$ plane $\rho_{\xi=0} - \rho_\infty = (\rho_{\rm sat} - \rho_\infty)R/(H_0 + a_0)$ (the subscript "0" denotes the initial value of the parameter). Because the tip is small compared to the drop we have $H_0 + a_0 \approx R$ and $\rho_{\rm sat} \approx \rho_{\xi=0}$. As a result, j_n is extremely sensitive to the exact value of $\rho_{\xi=0}$ and tiny errors, for instance introduced by our simple matching procedure, results in appreciable differences for the evaporation rate. We thus introduce a parameter q to finely tune the value of the matching field around the predicted value:

$$\rho_{\xi=0} - \rho_\infty = q(\rho_{\rm sat} - \rho_\infty)R/(H_0 + a_0).$$

This free parameter should be close to 1.

It was proven by Ham [5] that hyperboloidal surfaces are shape preserving solutions of the time dependent diffusion equation both during growth and shrinkage provided $\rho_{\xi=0} = \text{cst}$ (though the solution provided has no closed form). This is not strictly the case here as the drop shrinks with $R(t) = \sqrt{R_0^2 - 2\beta t}$ where $\beta = D\left(\rho_{\rm sat} - \rho_\infty\right)/\rho_{\rm ice}$ is an evaporation parameter including all the thermodynamic quantities. Though the evaporation being faster at the tip, this is a reasonable approximation at very short time close to the singularity, explaining the self-similar shapes observed in the experiments. The tip is thus characterized by only one parameter whose evolution is given by evaluating Supplementary Equation (1) at its apex ($\eta = 1$). Coming back to the usual hyperboloids parameters ($\xi_{\rm s} = a/c$) we get

$$\frac{\mathrm{d}a}{\mathrm{d}t} = \frac{2\beta a_0 c_0}{a(t) b_0^2 \ln\left(\frac{c_0+a_0}{c_0-a_0}\right)}\left(1 - q\frac{R(t)}{H_0 + a_0}\right). \tag{2}$$

Because our tips are initially very sharp ($a_0 \sim 1$ μm while $H_0 \sim 1$ mm) $H_0 + a_0 \approx H_0$ and integrating Supplementary Equation (2) between 0 and t we obtain

$$a(t)^2 - a_0^2 = \frac{4\beta a_0 c_0}{b_0^2 \ln\left(\frac{c_0+a_0}{c_0-a_0}\right)} \int_0^t \left(1 - q\frac{\sqrt{R_0^2 - 2\beta t^*}}{H_0}\right) \mathrm{d}t^*$$

$$a(t) = \sqrt{a_0^2 + \frac{4\beta a_0 c_0}{b_0^2 \ln\left(\frac{c_0+a_0}{c_0-a_0}\right)}\left[t + q\frac{R(t)^3 - R_0^3}{3\beta H_0}\right]}. \tag{3}$$

From Supplementary Equation (3) we finally calculate the tip curvature $\kappa(t) = 1/(a(t)\tan^2\alpha)$:

$$\kappa(t) = \left(\kappa_0^{-2} + \frac{4\beta\tan^2\alpha}{\ln\left(\frac{1+\cos\alpha}{1-\cos\alpha}\right)\cos\alpha}\left[t + q\frac{R(t)^3 - R_0^3}{3\beta H_0}\right]\right)^{-1/2}. \tag{4}$$

If we look close to the singularity, the variation of the outer field can be neglected for $t << R_0^2/2\beta$ and Supplementary Equation (4) simplifies to the pure $-1/2$ power law presented in the main text with the prefactor

$$A = \left(\frac{4\beta\tan^2\alpha}{\ln\left(\frac{1+\cos\alpha}{1-\cos\alpha}\right)\cos\alpha}\left[1 - q\frac{R_0}{H_0}\right]\right)^{-1/2}. \tag{5}$$

To test our model and quantify the error introduced by our simple boundary layer approach, we perform 2D axisymmetric finite element simulations of Eqs. (1) (2) (see Methods) for a perfect hyperboloid on a hemisphere where all the parameters are known precisely (except q). Supplementary Figure 1C shows the simulated equipotentials of $\rho(\mathbf{r})$. Like in the model, they are hyperboloidal close to the tip and spherical far from the drop. However, the field around the matching plane $\xi = 0$ is as expected more complex than in our simple approach. At short time, the simulated tip is self-similar like in the experiments and its radius of curvature is plotted in Supplementary Figure 2A along with Equation (3) and the full model Supplementary Equation (4). Close to the singularity, we recover as expected the $-1/2$ power law from the main text while at later time the deviation is well captured by our full model which takes the time variation of the outer field into account. The only free parameter here is q which we have adjusted to $q = 1.059$ corresponding to a tiny adjustment of 5.9% of the matching field. When the tip curvature

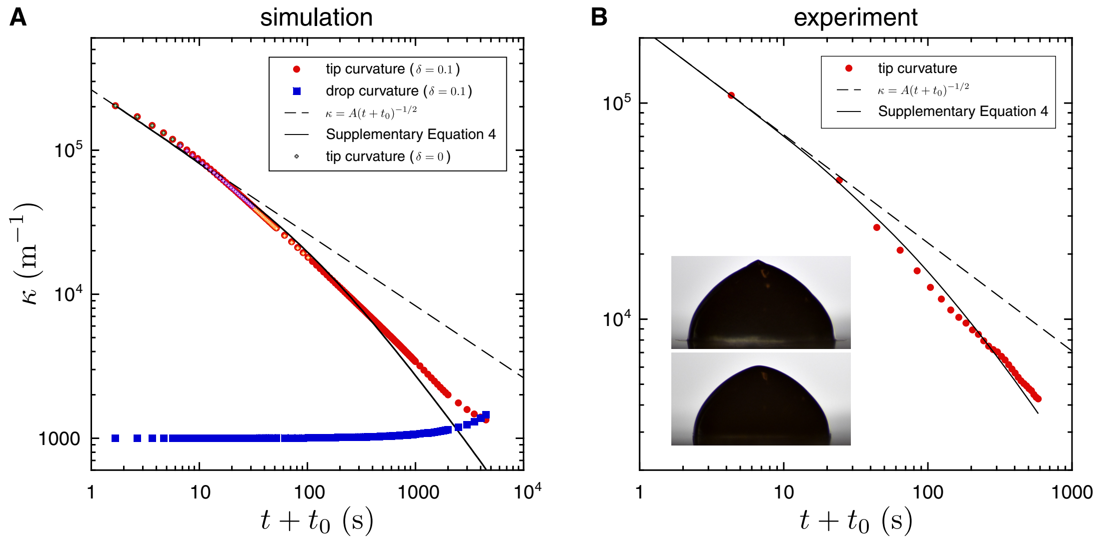

Supplementary Figure 2. **Tip smoothing on hemisphere model vs simulation and experiments. A** Curvature of a simulated hyperboloidal tip on an hemisphere (with and without the moving boundary smoothing option, $T_s = -10°$C, RH = 0%, $\alpha = 67.5°$, $R_0 = 1$ mm, $H_0 = 1.06$ mm). The solid and dashed lines corresponds to the model with $q = 1.059$. **B** Experimental tip curvature for a small, rapidly evaporating drop ($R_0 = 0.79$ mm, $H_0 = 0.86$ mm, $T_s = -4°$C, RH = 5.0%, $\alpha = 66°$, $\theta = 81°$). The solid and dashed lines corresponds to the model with $q = 1.075$. Inset: Pictures of the drop for the first and last data point.

becomes of the order of the drop curvature, the self-similarity gradually breaks down and even the full model starts to deviate from the simulation. Finally the tip curvature reaches the drop curvature (the inverse of its radius) which indicates its complete smoothing.

We use the moving boundary smoothing option (with $\delta = 0.1$) in the simulation to keep a good quality mesh during the deformations (see Methods). To check that it did not influence the smoothing observed, we ran the simulation of Supplementary Figure 2A with this parameter turned off ($\delta = 0$). The mesh degraded faster and we needed to remesh manually three times to simulate the first 100 s of evaporation. However, the results (shown in Supplementary Figure 2A) are identical confirming that this option does not influence the simulation outcome.

Now coming back to the experiments, because the domain of validity of the $-1/2$ power law ($t << R_0^2/2\beta$) depends on the experimental parameters, we either observe the pure power law as in Figure 4 for low substrate temperature (corresponding to low β) or also its deviation for higher substrate temperatures (corresponding to high β) as illustrated in Supplementary Figure 2B. Nonetheless, the full model taking into account the variation of the outer field is able to capture most of the smoothing for all our drops (with $q \sim 1$).

Matching with a spherical cap : On most surfaces, drops have contact angles deviating significantly from $\theta = 90°$, it is then inaccurate to assume that the tip sits on a hemisphere. Therefore, the matching should be done with the outer field of a spherical cap. The exact concentration field produced by a spherical cap is [6, 7]:

$$\rho = \rho_\infty + (\rho_{\mathrm{sat}} - \rho_\infty)\sqrt{2\left(\cosh\alpha^* - \cos\beta^*\right)}\int_0^\infty \frac{\cosh(\theta\tau)\cosh((2\pi-\beta^*)\tau)}{\cosh(\pi\tau)\cosh((\pi-\theta)\tau)} P_{-1/2+i\tau}(\cosh\alpha^*)\mathrm{d}\tau \tag{6}$$

with $P_{-1/2+i\tau}$ the (real-valued) Legendre functions of the first kind and (α^*,β^*) toroidal coordinates which are related to the cylindrical ones by:

$$r = \frac{R\sinh\alpha^*}{\cosh\alpha^* - \cos\beta^*}, \qquad z = \frac{R\sin\beta^*}{\cosh\alpha^* - \cos\beta}.$$

Although, as for the hemisphere, this field is not flat on the $\xi = 0$ plane, we will neglect its curvature and take its value on $r = 0$. On this line the toroidal coordinates simplifies, $\alpha^* = 0$ such that $P_{-1/2+i\tau}(\cosh\alpha^*) = P_{-1/2+i\tau}(1) = 1$ and $\beta^* = 2\arctan(R/z) + 2k\pi$ with k a natural number. To simplify the integral we use $k = 1$ [8] such that

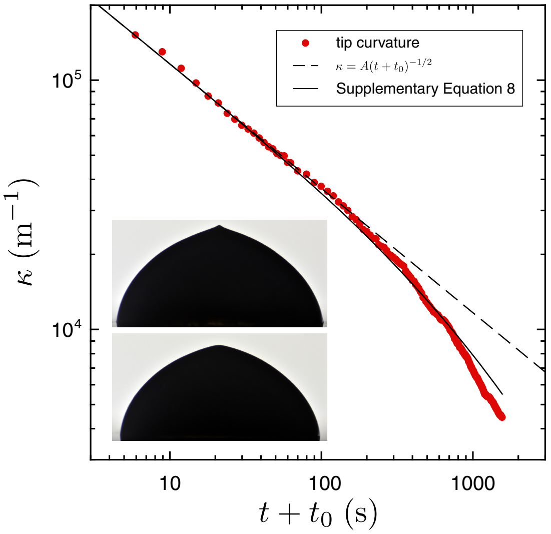

Supplementary Figure 3. **Tip smoothing on spherical cap model vs experiments.** Experimental tip curvature for a drop evaporating at moderate speed ($R_0 = 1.20$ mm, $H_0 = 1.12$ mm, $T_s = -10°$C, RH = 4.7%, $\alpha = 64.9°$, $\theta = 79°$). The solid and dashed lines corresponds to the model with $q = 1.075$. Inset: Pictures of the drop for the first and last data point.

Supplementary Equation (6) simplifies to

$$\rho(r=0,z) = \rho_\infty + 2(\rho_{\text{sat}} - \rho_\infty)\frac{R}{\sqrt{R^2+z^2}}\int_0^\infty \frac{\cosh(\theta\tau)\cosh(2\arctan(R/z)\,\tau)}{\cosh(\pi\tau)\cosh((\pi-\theta)\tau)}\mathrm{d}\tau.$$

The integral has no closed-form solution in the general case (for $\theta = \pi/2$, however, we recover the hemisphere solution) but since the tip is small compared to the drop, and since we want to evaluate the concentration at the tip, we simplify it by assuming $z \approx h_{\text{cap}} = R(1-\cos\theta)/\sin\theta$, with h_{cap} the height of a spherical cap. This results in $2\arctan(R/z) \approx \pi - \theta$ which greatly simplifies the integral with an error on the final field around 10% for typical values of R and $H_0 + a_0$. The final matching field is thus

$$\rho_{\xi=0} - \rho_\infty = 2q(\rho_{\text{sat}} - \rho_\infty)\frac{R}{\sqrt{R^2+(H_0+a_0)^2}}\int_0^\infty \frac{\cosh(\theta\tau)}{\cosh(\pi\tau)}\mathrm{d}\tau.$$

Here, we have also introduced the free parameter q for the previous mentioned reasons. After this simplification, the procedure is identical to the one for hemispheres and we get

$$\frac{\mathrm{d}a}{\mathrm{d}t} = \frac{2\beta a_0 c_0}{a(t) b_0^2 \ln\left(\frac{c_0+a_0}{c_0-a_0}\right)}\left(1 - \frac{q f(\theta)}{\sqrt{1+\left(\frac{H_0+a_0}{R(t)}\right)^2}}\right) \tag{7}$$

$$R(t) = \sqrt{R_0^2 - 2\beta t g(\theta)}$$

with $f(\theta) = 2\int_0^\infty \frac{\cosh(\theta\tau)}{\cosh(\pi\tau)}\mathrm{d}\tau$ and

$$g(\theta) = \left(\frac{\sin(\theta)^3}{2-3\cos(\theta)+\cos(\theta)^3}\right)\left(\frac{\sin\theta}{1+\cos\theta} + 4\int_0^\infty \frac{1+\cosh(2\theta\tau)}{\sinh(2\pi\tau)}\tanh((\pi-\theta)\tau)\mathrm{d}\tau\right).$$

To compute $g(\theta)$ we have used Equations (A8) and (A9) from Popov [9] and assumed the matching field to vary like that of drops evaporating in the constant contact angle mode. Supplementary Equation (7) can then be solved

analytically to obtain the following equation for the tip curvature:

$$\kappa(t) = \left(\kappa_0^{-2} + \frac{4\beta \tan^2 \alpha}{\ln\left(\frac{1+\cos\alpha}{1-\cos\alpha}\right)\cos\alpha} \left[t + \frac{q f(\theta)}{2\beta g(\theta)} \omega(t) \right] \right)^{-1/2} \quad \text{with}$$
$$\omega(t) = R(t)\sqrt{R(t)^2 + H_0^2} - R_0\sqrt{R_0^2 + H_0^2} - H_0^2 \ln\left(\frac{R(t) + \sqrt{R(t)^2 + H_0^2}}{R_0 + \sqrt{R_0^2 + H_0^2}} \right). \quad (8)$$

We have also dropped a_0 here since it is negligible compared to H_0. Again, close to the singularity the variation of the outer field can be neglected for $t << R_0^2/(2\beta g(\theta))$ and Supplementary Equation (8) simplifies to the pure $-1/2$ power law presented in the main text with the prefactor

$$A = \left(\frac{4\beta \tan^2 \alpha}{\ln\left(\frac{1+\cos\alpha}{1-\cos\alpha}\right)\cos\alpha} \left[1 - q \frac{f(\theta) R_0}{\sqrt{R_0^2 + H_0^2}} \right] \right)^{-1/2}. \quad (9)$$

For the drop presented in Figure 4cd which has a contact angle of $\theta = 95.5°$ and a tip angle of $\alpha = 69.8°$ we can use Supplementary Equation (9) to calculate the prefactor A. We recover the fitted value $A = 2.32 \cdot 10^5$ $\mathrm{s}^{1/2}\,\mathrm{m}^{-1}$ with $q = 1.058$, i.e. an adjustment of 5.8% of the matching field. If we do not correct the matching field (setting $q = 0$) we get $A = 1.05 \cdot 10^5$ $\mathrm{s}^{1/2}$ m^{-1}, a factor two error which illustrate the sensitivity of A on the precise value of matching field. Like in the hemispherical case, deviations to the $-1/2$ power law are visible for rapidly evaporating drops. We show in Supplementary Fig 3 a drop evaporating moderately fast which exhibits clearly the two regimes. Also here the full model captures the full smoothing process with $q \approx 1$.

SUPPLEMENTARY NOTE 2: LATE TIME EVAPORATION MODEL

While the tip of the ice drop gets smoothed, a similar phenomenon occurs at the drop edge. Supplementary Figure 4A shows the radius, height and apparent contact angle of the initially supercooled drop that subsequently freeze shown in Supplementary Movie 3. In the liquid state, the drop evaporates with a strongly pinned contact line in the constant contact radius mode [10]: its height and contact angle decrease while its radius remains constant. For the frozen drop, on the other hand, both the height and radius decrease similarly to a liquid drop evaporating constant contact angle mode. Yet, the apparent contact angle of the ice drop is far from constant: it rapidly increases towards a plateau around $\theta \sim 90°$. The drop edge gets smoothed similarly to the drop tip because of its high curvature which result in an increase of the apparent macroscopic contact angle. The geometry is quite similar to hyperboloidal tip

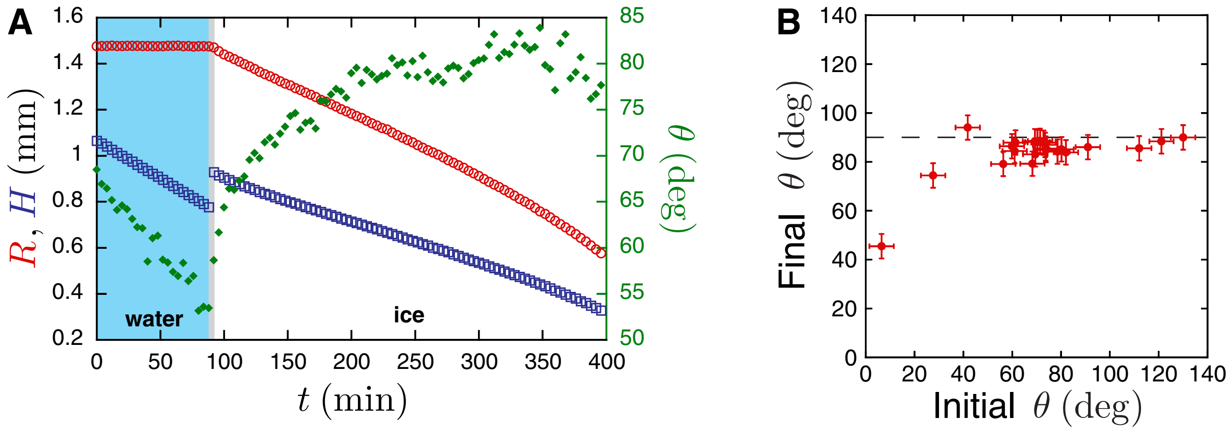

Supplementary Figure 4. **Ice drop shape evolution. A** Radius (red circles), height (blue squares) and apparent contact angle (green diamonds) of the drop presented in Figure 3 (supercooled until $t = 92$ min, $T_s = -15°$C, RH = 2.8%, see Supplementary Movie 3). **B** Initial liquid drop contact angle vs late time ice drop contact angle. The error bars are s.e.m. and the black dashed line is $\theta = 90°$.

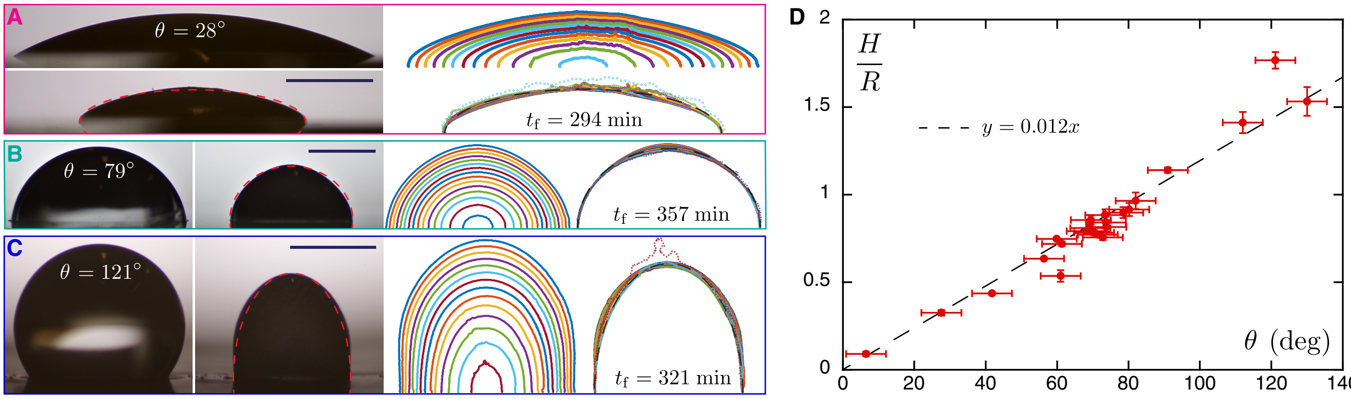

Supplementary Figure 5. **Late time drop shape.** **A-C** Pictures of the initial water drop and the subsequent ice drop at $t = t_f/2$ with an ellipse superimposed (red dashed line) for drops with identical parameters except the wettability ($V_0 \approx 3.9$ μL, $T_s = -10$°C, RH $\approx$ 5%), scale bars 1 mm. The corresponding experimental profiles for $t > t_f/2$ (12-min increments) and the same profiles rescaled by the drop radii with an ellipse superimposed (black dashed line) are shown. **D** Aspect ratio H/R at late time ($t > t_f/2$) as a function of the initial water drop contact angle. The horizontal error bars are s.e.m., the vertical ones s.d. and the dashed line is a linear fit.

but in two dimensions. Indeed, neglecting the smaller outer plane curvature, the drop edge can be assimilated as half of a sharp corner which get smoothed in an hyperbola shape with a decreasing curvature.

After about half of the total evaporation time t_f all the sharp features of our pointy drops have disappeared and they reach a $\approx 90°$ contact angle (see Supplementary Figure 4B). As for liquid droplets, repeating the experiment with a different substrate temperature or humidity only impacts the speed of the evaporation, but not the late time shape evolution of the drop. Supplementary Figure 5ABC shows pictures of the initial water drop as well as the frozen drop at $t_f/2$ and the subsequent drop profiles for $t > t_f/2$ but for drops with different initial shapes (only the substrate wettability is varied, the other parameters are kept constant). All drops evolve toward self-similar spheroidal shapes at later time with an aspect ratio which depends on the initial liquid drop contact angle θ. Moreover, the drop lifetime t_f (and thus its evaporation rate) depends non-monotonically on the wettability, the slowest drop being the closest to a hemisphere with a lifetime $\approx$ 20% higher than the fastest one. We plot in Supplementary Figure 5D the late-time aspect ratio as a function of the initial contact angle for all our experiments (T_s, RH, V_0 and θ are varied). The aspect ratio of the smoothed, spheroidal ice drop only depends on the contact angle of the water drop when it freezes and the relationship appears to be linear over the measurement range.

Ham [5] has shown that ellipsoids (and thus spheroids) are shape-preserving solutions of the time-dependent diffusion-limited growth (though the solution provided has no closed form). However, unlike the hyperboloidal case, the proof is not valid for shrinking surfaces. Though, the self-similar shapes presented in Supplementary Figure 5ABC suggest this is also true in that case. We further checked the self-similarity with finite element simulations of sublimating ellipsoids. We show in Supplementary Figure 6 the profiles of spheroids and triaxial ellipsoids. They all collapse on the initial profile once rescaled by the major axis, confirming that ellipsoids, in general, are also shape-preserving solutions of Eqs. (1)(2) in the shrinking case.

Here also the self-similarity allows to solve the problem analytically and Laplace's equation (1) can be solved for ellipsoids using ellipsoidal coordinates. From the derivative of the field we can compute the local volume flux [6]. Calling a, b, c the three semi-axes of this ellipsoid (such that $a > b > c$) we get

$$j_n = \frac{\beta C^{\text{tri}}}{abc\sqrt{\frac{x^2}{a^4}+\frac{y^2}{b^4}+\frac{z^2}{c^4}}} \tag{10}$$

where C^{tri} is the triaxial ellipsoid capacitance given by [6, 11]:

$$\frac{1}{C^{\text{tri}}} = \frac{1}{2}\int_0^\infty \frac{\mathrm{d}s}{\sqrt{(a^2+s)(b^2+s)(c^2+s)}} = \frac{F_1\left(\frac{1}{2},\frac{1}{2},\frac{1}{2},\frac{3}{2},1-(\frac{b}{a})^2,1-(\frac{c}{a})^2\right)}{a} \tag{11}$$

with F_1 the Appell hypergeometric function. Then, because the ellipsoid keeps an ellipsoidal shape at all time, it can be fully described by just its semi-axes whose evolution is given by Equation (2) applied on its three edges. We

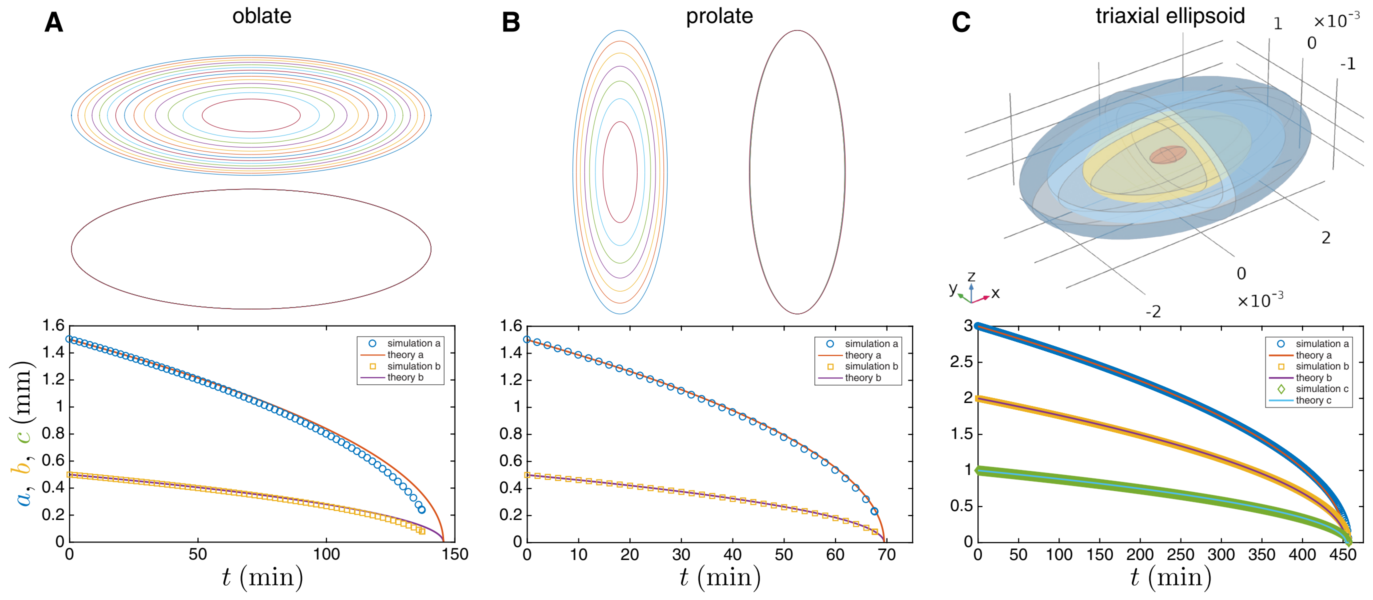

Supplementary Figure 6. **Ellipsoid evaporation model vs simulations.** **A** Raw and rescaled profiles of an evaporating/dissolving simulated oblate spheroid (10-min increments) and evolution of its semi-axes as a function of time. Same data for **B** a prolate spheroid (5-min increments) and **C** a triaxial ellipsoid (150-min increments; a portion of the surface is hidden for visualization), $\beta = 5.6\,10^{-11}$ m^2 s^{-1} for all the simulations.

obtain the coupled first order system

$$\frac{\mathrm{d}a}{\mathrm{d}t} = -\frac{\beta C^{\mathrm{tri}}(t)}{b(t)c(t)}, \qquad \frac{\mathrm{d}b}{\mathrm{d}t} = -\frac{\beta C^{\mathrm{tri}}(t)}{a(t)c(t)}, \qquad \frac{\mathrm{d}c}{\mathrm{d}t} = -\frac{\beta C^{\mathrm{tri}}(t)}{a(t)b(t)} \tag{12}$$

which we solve (the subscript "0" denotes the initial value of the parameter)

$$a(t) = \sqrt{a_0^2 - 2\beta\left(a_0 C_0^{\mathrm{tri}}/b_0 c_0\right) t}, \qquad b(t) = (b_0/a_0)\, a(t), \qquad c(t) = (c_0/a_0)\, a(t). \tag{13}$$

The special case of spheroids corresponds to $b = c$ for prolate spheroids and $a = b$ for oblate ones (spheres to $a = b = c$) and greatly simplifies the capacitance to

$$C^{\mathrm{ob}} = \frac{\sqrt{a^2 - b^2}}{\arcsin\left(\sqrt{1-(b/a)^2}\right)}, \qquad C^{\mathrm{pro}} = \frac{2\sqrt{a^2-b^2}}{\ln\left(\frac{a+\sqrt{a^2-b^2}}{a-\sqrt{a^2-b^2}}\right)}.$$

We first test our model with the simulated ellipsoids and spheroids to Supplementary Equation (13) in Supplementary Figure 6 and find a very good agreement. From Supplementary Equation (13) we can then deduct several measurable quantities such as the ellipsoid volume $V(t)$, the evaporation time t_{f} or the global evaporation flux $\mathrm{d}V(t)/\mathrm{d}t$

$$V(t) = \frac{4}{3}\pi \frac{b_0 c_0}{a_0^2} a(t)^3 \sim (t_{\mathrm{f}} - t)^{3/2}, \qquad t_{\mathrm{f}} = \frac{a_0 b_0 c_0}{2\beta C_0^{\mathrm{tri}}}, \qquad \frac{\mathrm{d}V}{\mathrm{d}t} = -4\pi\beta\left(C_0^{\mathrm{tri}}/a_0\right) a(t).$$

Now, to compare our spheroid model to experimental data, a and b represent the ice drop height and radius (or radius and height depending on the aspect ratio). Supplementary Equation (13) is thus equivalent for our drop to

$$R(t) = \sqrt{R_0^2 - 2\beta\left(C_0^{\mathrm{ob/pro}}/H_0\right) t}, \qquad H(t) = (H_0/R_0)\, R(t). \tag{14}$$

We measure the drop radius and height at $t = t_{\mathrm{f}}/2$, the beginning of the late time regime, and use these parameters as initial values for Supplementary Equation (14).

The results of the model (also back propagated for $t < t_{\mathrm{f}}/2$) is shown in Figs. 3 and 5ab. The model agrees very well with experiments for $t > t_{\mathrm{f}}/2$ and captures the complete shape evolution. For $t < t_{\mathrm{f}}/2$, the agreement is less satisfactory, especially at the beginning of the experiment, when the drop is far from being spheroidal.

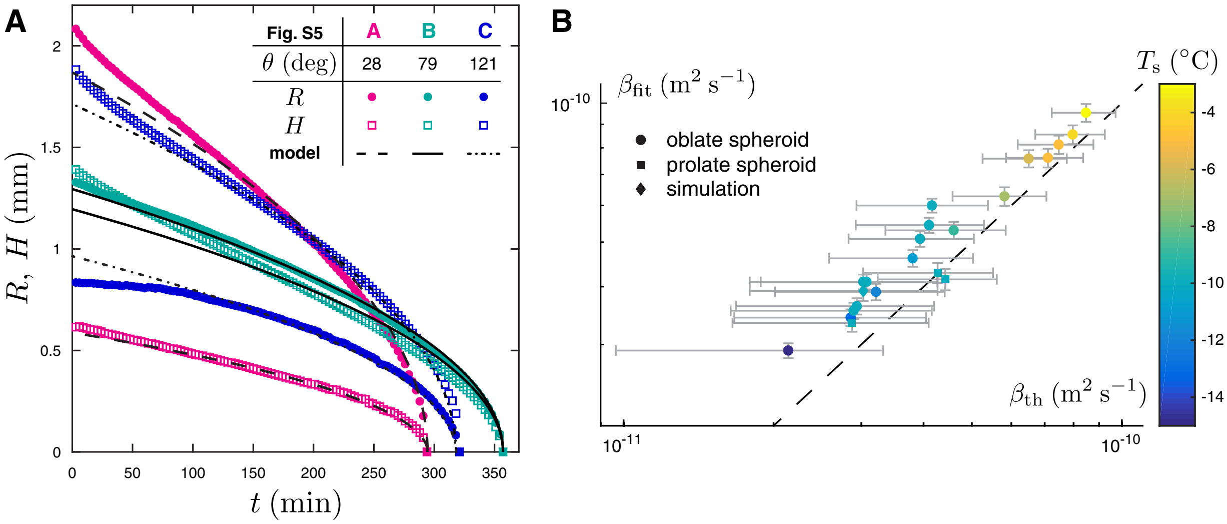

Supplementary Figure 7. **Global ice drop evaporation.** **A** Ice drop height H and radius R as a function of time for the drops shown in Supplementary Figure 6. The lines are the results of the spheroid model (Supplementary Equation (14)) front and back propagated taking $t_f/2$ as initial conditions. **B** Comparison between the fitted and calculated evaporation parameters for all our experiments. The error bars are s.e.m. and the dashed line is $\beta_{\text{fit}} = \beta_{\text{th}}$.

As we have seen in Supplementary Figure 5ABC changing the substrate wettability changes the drop shape and its evaporation rate. We plot in Supplementary Figure 7A the experimental radius R and height H of these ice drops and compare them to our spheroid model. Again good agreement is found at late time, showing that the model captures the non trivial shape dependence on the evaporation rate.

Although the late time model is parameter free, we had to slightly adjust the evaporation rate $\beta = D\left(\rho_{\text{sat}} - \rho_\infty\right)/\rho_{\text{ice}}$ within the experimental uncertainties to obtain this excellent agreement with the experiments. The same goes for the simulation presented in Supplementary Fig. 5. Supplementary Figure 7B shows the fitted vs calculated evaporation parameter for all our experiments (T_s, RH, V_0 and θ are varied). The fitted and calculated values of β agree within the experimental uncertainties, which are significant at low β (the horizontal error bars are calculated by propagating the uncertainties on the plate temperature $\Delta T_s = 0.2°\text{C}$ and the humidity $\Delta\text{RH} = 2\%$). Nonetheless, our calculated evaporation rate is systematically lower than the one we fit. This probably come from the neglected thermal gradients (the drop being slightly hotter than T_s) or the small convection contribution (from Stefan flow or the nitrogen injection).

Finally, because the problem is mathematically equivalent to the diffusion limited dissolution of solids, our theory is also valid in that situation and our results could be used to model drug dissolution in quiescent solutions. A pill of any shape put into solution will quickly lose any sharp edge or point like our pointy drops and reach a smooth ellipsoidal shape close to its initial aspect ratio. We can thus model its dissolution with Supplementary Equation (13) and predict the amount of drug released at time t or the total dissolution time

In Supplementary Figure 8A we show the effect of the pill shape on its dissolution speed, plotting the dimensionless dissolution time as a function of the ellipsoid aspect ratios (Supplementary Figure 8B shows the special cases of spheroids). For a given pill volume V_0 and a given solvent and solute (fixing β), the slowest pill to dissolve is the spherical one. Up to aspect ratios 1:2 the ellipsoidal pills dissolve almost as slowly as the sphere but for higher aspect ratio the difference becomes appreciable, with elongated pills (aspect ratios $<<1$) dissolving much faster.

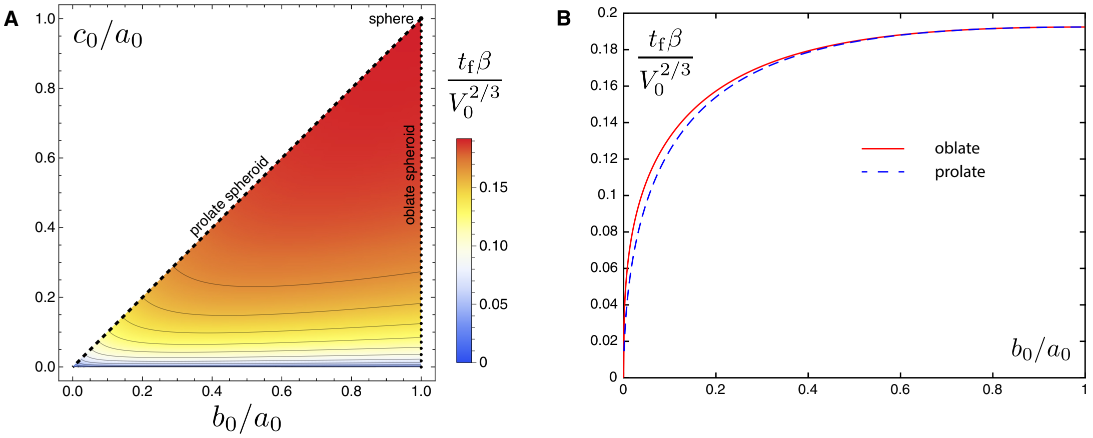

Supplementary Figure 8. **Dissolution time of ellipsoids.** **A** Dimensionless dissolution time for a triaxial ellipsoid as a function if its aspect ratios. The special cases of the sphere ($a_0 = b_0 = c_0$), prolate ($a_0 = b_0 > c_0$) and oblate ($a_0 > b_0 = c_0$) spheroids are highlighted. **B** Dimensionless dissolution time for spheroids.

SUPPLEMENTARY REFERENCES

[1] Lekner, J. Comparison of hyperbolic and hyperboloid conductor electrostatics. *Eur. J. Phys.* **27**, 87 (2006).

[2] Yarin, A. L., Koombhongse, S. & Reneker, D. H. Taylor cone and jetting from liquid droplets in electrospinning of nanofibers. *J. Appl. Phys.* **90**, 4836–4846 (2001).

[3] Weisstein, E. W. Prolate spheroidal coordinates. From MathWorld—A Wolfram Web Resource. `http://mathworld.wolfram.com/ProlateSpheroidalCoordinates.html`. Accessed: 2018-07-16.

[4] Eyring, C. F., Mackeown, S. S. & Millikan, R. A. Fields currents from points. *Phys. Rev.* **31**, 900–909 (1928).

[5] Ham, F. S. Shape-preserving solutions of the time-dependent diffusion equation. *Quart. Appl. Math.* **17**, 137–145 (1959).

[6] Lebedev, N. N., Skalskaya, I. P. & Uflyand, Y. S. *Problems in mathematical physics* (Pergamon, 1966).

[7] Deegan, R. D. *et al.* Contact line deposits in an evaporating drop. *Phys. Rev. E* **62**, 756–765 (2000).

[8] Carrier, O. *et al.* Evaporation of water: evaporation rate and collective effects. *J. Fluid Mech.* **798**, 774786 (2016).

[9] Popov, Y. O. Evaporative deposition patterns: Spatial dimensions of the deposit. *Phys. Rev. E* **71**, 036313 (2005).

[10] Picknett, R. & Bexon, R. The evaporation of sessile or pendant drops in still air. *J. Colloid Interface Sci.* **61**, 336 – 350 (1977).

[11] Kraniotis, G. V. & Leontaris, G. K. Closed form solution for the surface area, the capacitance and the demagnetizing factors of the ellipsoid (2013). arXiv:1306.0509.
