# Supplementary material for: Singular sublimation of ice and snow crystals
Source: Nat Commun. 2018 Oct 10;9:4191. doi: 10.1038/s41467-018-06689-x (PMC6180084; doi:10.1038/s41467-018-06689-x)
Supplement: Supplementary file 3 — Description of Additional Supplementary Files [file 41467_2018_6689_MOESM3_ESM.pdf]

Description of Additional Supplementary Files

File Name: Supplementary Movie 1
Description: Simulation of the evaporating snowflake corresponding to Fig. 1b

File Name: Supplementary Movie 2
Description: Evaporation of the pointy ice drop corresponding to Fig. 2 and Fig. 5

File Name: Supplementary Movie 3
Description: Comparison between liquid water and solid ice evaporation, experiment corresponding to Fig. 3

File Name: Supplementary Movie 4
Description: Pointy drop tip smoothing, experiment corresponding to Fig. 4ab
